# Atrial fibrillation risk in athletes: it's not the sport, it's the mileage

**DOI:** 10.1093/ehjopen/oeag090

**Published:** 2026-06-03

**Authors:** Marius Myrstad, Eirik Mølmshaug, Kristoffer Robin Johansen

**Affiliations:** Department of Medical Research, Bærum Hospital Vestre Viken Hospital Trust, Sogneprest Munthe-Kaasvei 100, N-1346 Gjettum, Norway; Department of Medical Research, Bærum Hospital Vestre Viken Hospital Trust, Sogneprest Munthe-Kaasvei 100, N-1346 Gjettum, Norway; Faculty of Medicine, University of Oslo, Postboks 6706 St. Olavs plass, N-0130 Oslo, Norway; School of Sport Sciences, UiT the Arctic University of Norway, PO Box 6050 Stakkevollan, N-9037 Tromsø, Norway; Centre for Research and Education, University Hospital of North Norway, PO Box 100, N-9038 Tromsø, Norway

**This editorial refers to ‘Association of Self-reported Sports Volume and Discipline with Atrial Arrhythmia Prevalence in Middle-Aged Males’, by J. De Paepe *et al.*, https://doi.org/10.1093/ehjopen/oeag089.**

Endurance exercise increases the risk of atrial fibrillation (AF), but despite improved insight from observational studies over the past 25 years, tools for AF risk stratification in athletes are lacking.^1^ De Paepe and colleagues studied the association between endurance exercise and risk of AF and atrial flutter (AFL) in a cross-sectional analysis among 3939 healthy men aged >45 years screened for participation in the Master@Heart study. Atrial fibrillation/AFL and exercise were self-reported by questionnaires, and lifetime exercise hours were estimated (weekly hours × years of exercise).^2^ Using a similar design as previous studies, the authors report an increased AF risk among the men with the highest cumulative training load, with adjusted odds ratios (aORs) for AF/AFL around 2.0.

This relative risk estimate is strikingly similar to previous studies with an approximate two-fold risk increase for AF reported among middle-aged and older male athletes (*Figure 1B*). In the Birkebeiner study that included 2366 non-elite cross-country (XC)-skiers, the aOR for ECG-validated AF/AFL was 1.9 in the groups with the highest training load.^3^ Similarly, in another study including 509 XC-skiers, the aOR for self-reported AF was 1.9.^4^ Both the Master@Heart study and the Birkebeiner study found a gradual increase in AF/AFL risk by exercise, with 2% increase/1000 exercise hours and 16% increase/10 years of exercise, respectively.

**Figure 1:**
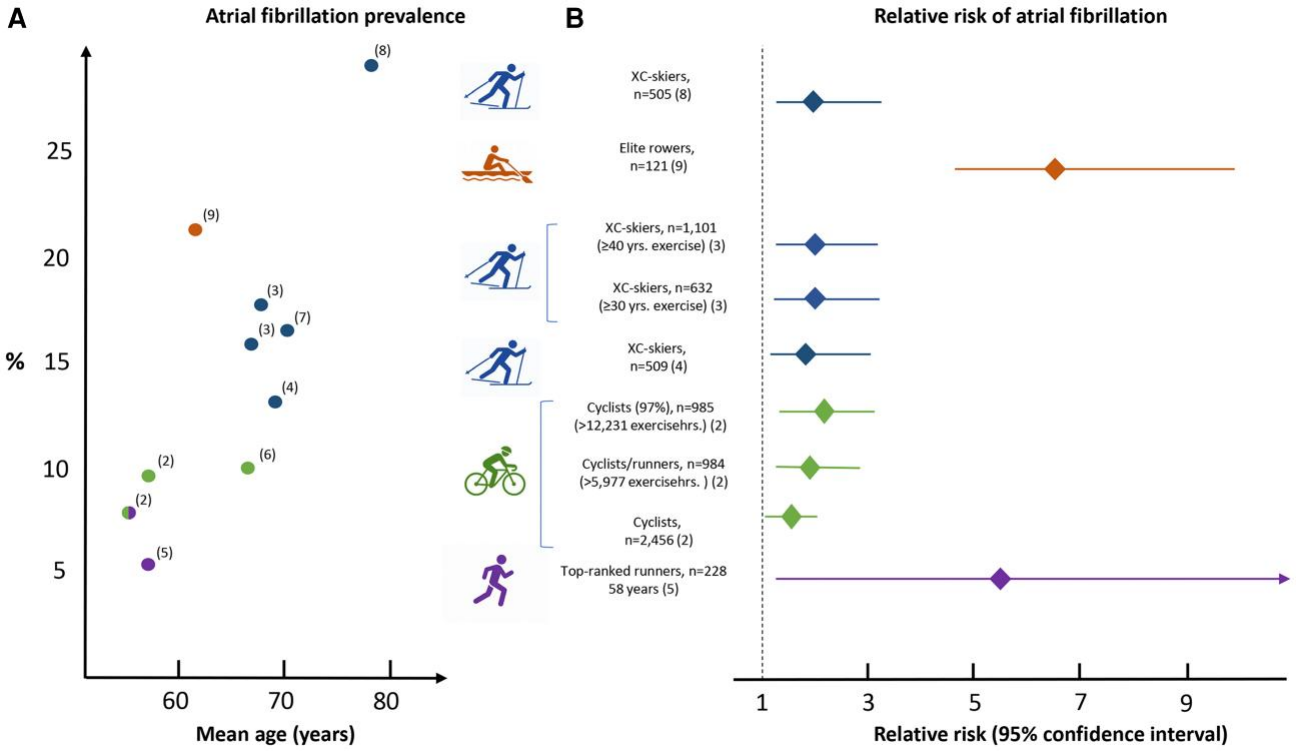
(*A*) Prevalence of atrial fibrillation (AF) plotted against mean age of study cohorts including XC-skiers (blue), cyclists (green), runners (violet), and rowers (orange). (*B*) Relative risk of AF with 95% confidence interval.

De Paepe and colleagues report an overall prevalence of AF/AFL of 7.5%. When combining the results of cohort studies, it becomes apparent that the prevalence of AF increases with age, from 5% to 10% in runners and cyclists aged 55–60 years to 10–18% in cyclists and skiers aged 65–70 years^2–7^ (*Figure 1A*). The highest prevalence of 28.5% has been reported in XC-skiers with a mean age of almost 80 years.^8^ However, a recently published study including 121 former elite rowers (mean age 62 years) reported an AF prevalence of 21.5%, markedly higher than in previous cohorts of athletes with a similar mean age.^9^ Compared with a matched control group form the UK Biobank, the rowers had an almost 7-fold increased risk of AF.^9^ In the seminal case-control study by Karjalainen,^5^ a 5.5-fold increased risk of AF was observed among top-ranked runners (orienteers). These findings suggest that athletes that have been exposed to a very high cumulative training load, and by extension having achieved a very high fitness level, may have the highest risk of AF.

Based on the observation that cycling but not running or swimming was associated with an increased risk, De Paepe and colleagues suggest a potential sports-specific effect on AF/AFL. This hypothesis is intriguing, and the authors should be commended for their attempt to further decipher the determinants of the excess AF risk in athletes. The individual risk of developing AF in individuals engaged in endurance sports depends on multiple factors, including age, sex, stature, the presence of established cardiovascular risk factors, polygenic risk, degree of cardiac remodelling and cumulative exposure to endurance exercise, but it remains unclear which risk factors most decisively influence AF risk in athletes. Exercise duration, -intensity, and type of sports are highly interrelated and many athletes practice several sports disciplines. To overcome the challenge of separating effects of the different exercise characteristics on cardiac remodelling and arrhythmia risk, prospective studies with high quality exercise data are warranted. Although pooling data from multiple cohorts and applying novel analytical methods may provide further insight, efforts from other research disciplines are needed to advance our understanding of the physiological effects of different endurance sport disciplines. Based on the current knowledge, milage is more convincingly associated AF risk than sports discipline.

## Data Availability

The data underlying this article are available in the article and in the references.
